# Supplementary material for: Non-identical moiré twins in bilayer graphene
Source: Nat Commun. 2023 Dec 11;14:8178. doi: 10.1038/s41467-023-43965-x (PMC10713781; doi:10.1038/s41467-023-43965-x)
Supplement: Supplementary file 1 — Supplementary Information [file 41467_2023_43965_MOESM1_ESM.pdf]

Non-identical moiré twins in bilayer graphene Supplementary Information

Everton Arrighi[1,+], Viet-Hung Nguyen[2,+], Mario Di Luca[1], Gaia Maffione[1], Yuanzhuo Hong[1], Liam Farrar[1], Kenji Watanabe[3], Takashi Taniguchi[3], Dominique Mailly[1], Jean-Christophe Charlier[2], and Rebeca Ribeiro-Palau[1,*]

[1]Université Paris-Saclay, CNRS, Centre de Nanosciences et de Nanotechnologies (C2N), 91120 Palaiseau, France
[2]Institute of Condensed Matter and Nanosciences, Université catholique de Louvain (UCLouvain), 1348 Louvain-la-Neuve, Belgium
[3]National Institute for Materials Science, 1-1 Namiki, Tsukuba, Japan
[*]rebeca.ribeiro@c2n.upsaclay.fr
[+]these authors contributed equally to this work

Supplementary Note 1: Samples used in this manuscript

Sample I (H038): described in details in the the main text, it has a central graphite gate. In Supplementary Figure 1 we present three different crystallographic alignments of the BN in this sample: 0°, 30° and 60°. The characteristic dimensions are: $W = 1.7\ \mu$m, $L = 2.3\ \mu$m, and the thickness of the bottom BN is 24 nm. Capacitive coupling is $C_g/e = 6.4 \times 10^{11}$ V^{-1} cm^{-2} (Hall measurements).

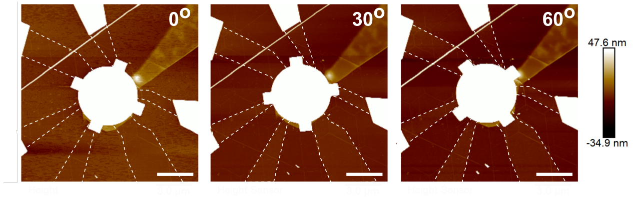

Supplementary Figure 1. Sample I. Atomic force microscopy in tapping mode at 0°, 30° and 60° of alignment. Dashed lines highlight the Hall bar shape of the graphene layer. Scale bar 3 μm.

Sample II (H012): it was built using the same techniques but instead of a local graphite gate it has a global graphite gate, Supplementary Figure 2. This sample is composed of two parts with two independent handles. For all the measurements presented here the BN handle in the left was misaligned. The characteristic dimensions are: $W = 1.8\ \mu$m, $L = 3\ \mu$m, bottom BN thickness of 55 nm. Capacitive coupling is $C_g/e = 2.7 \times 10^{11}$ V^{-1} cm^{-2} (Hall measurement).

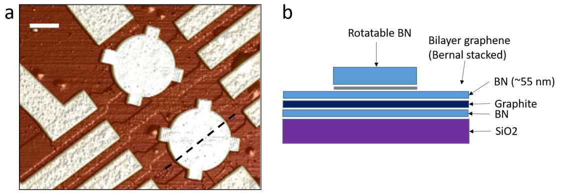

Supplementary Figure 2. Sample II. a Tapping mode AFM image of the sample. Scale bar 2.5 μm. **b**, Schematic cross-section of the heterostructure at the position of the black dashed line.

Sample III (MDL007a): it has the same structure as sample I and it has been used to characterize the alignment inside the AFM. The characteristic dimensions are: $W = 1.2\ \mu$m, $L = 1.5\ \mu$m, and bottom BN thickness of 50 nm. Capacitive coupling is $C_g/e = 3.76 \times 10^{11}$ V^{-1} cm^{-2} (plane capacitor calculation).

Sample IV (SBG06): with the same structure as sample I but different geometry. The characteristic dimensions are: $W = 2.3\ \mu$m, $L = 1.7\ \mu$m, and bottom BN thickness of 66 nm. Capacitive coupling $C_g/e = 3.02 \times 10^{15}$ V^{-1} m^{-2} (Hall measurement).

Supplementary Note 2: Crystallographic alignment characterization

As explained in the main text, we use an AFM tip to push the capping BN layer, by applying a force to one of the arms we are able to rotate it, as it can be seen in Supplementary Figure 1. As we approach the position where graphene and BN are crystallographically aligned, the resistance peak around the CNP becomes larger. This enlargement appears every sixty degrees, Supplementary Figure 3. However, we can see that the enlargement is not the same every sixty degrees, instead it has a hundred and twenty degrees periodicity, that can be seen even at room temperature. In Supplementary Figure 3a, b and c, we can clearly see the similarities between the curves: at 0° and 120° we observe similar height (R_{4P}^{CNP}) and shift on the voltage of the CNP ($V_{CNP} - V_{CNP}^{30°}$), while they are different from the ones at 60° and 180°.

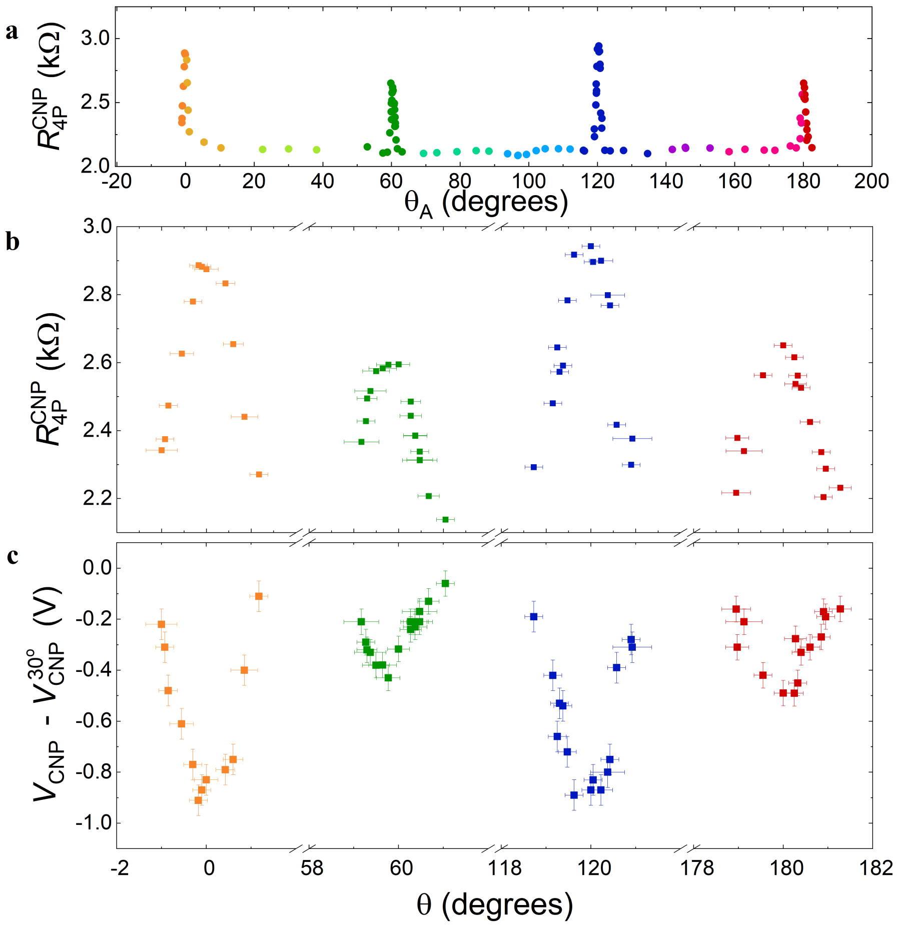

Supplementary Figure 3. Crystallographic alignment at room temperature sample III. a, Resistance of the CNP at as a function of the angular alignment, measured with the AFM, from -2° to 182°. **b**, Resistance value of the CNP as a function of the angular alignment around the aligned positions. **c**, Shift in voltage of the CNP, with respect to the misaligned position (30°), as a function of the angular alignment around around the aligned positions.

The periodicity of this behavior is observed in all the measured samples, see Supplementary Figure 3, 4 and Fig 2c of the main text. In all measurements the height of the resistance peak and the position of the CNP in voltage are periodic every hundred and twenty degrees.

It is important to highlight that the differences between 0° and 60° alignment becomes evident in our experiments only because we are able to measure the same sample with different crystallographic alignments. In other words, if we had two different samples with these characteristics, the difference would be attributed to sample-to-sample variation and not to a real effect of the angular alignment.

In an intuitive picture we can expect that if both aligned positions are obtained in the same sample and share about the same properties at room temperature the difference between them is coming from a more subtle difference, a different effective mass (see discussion in the main text).

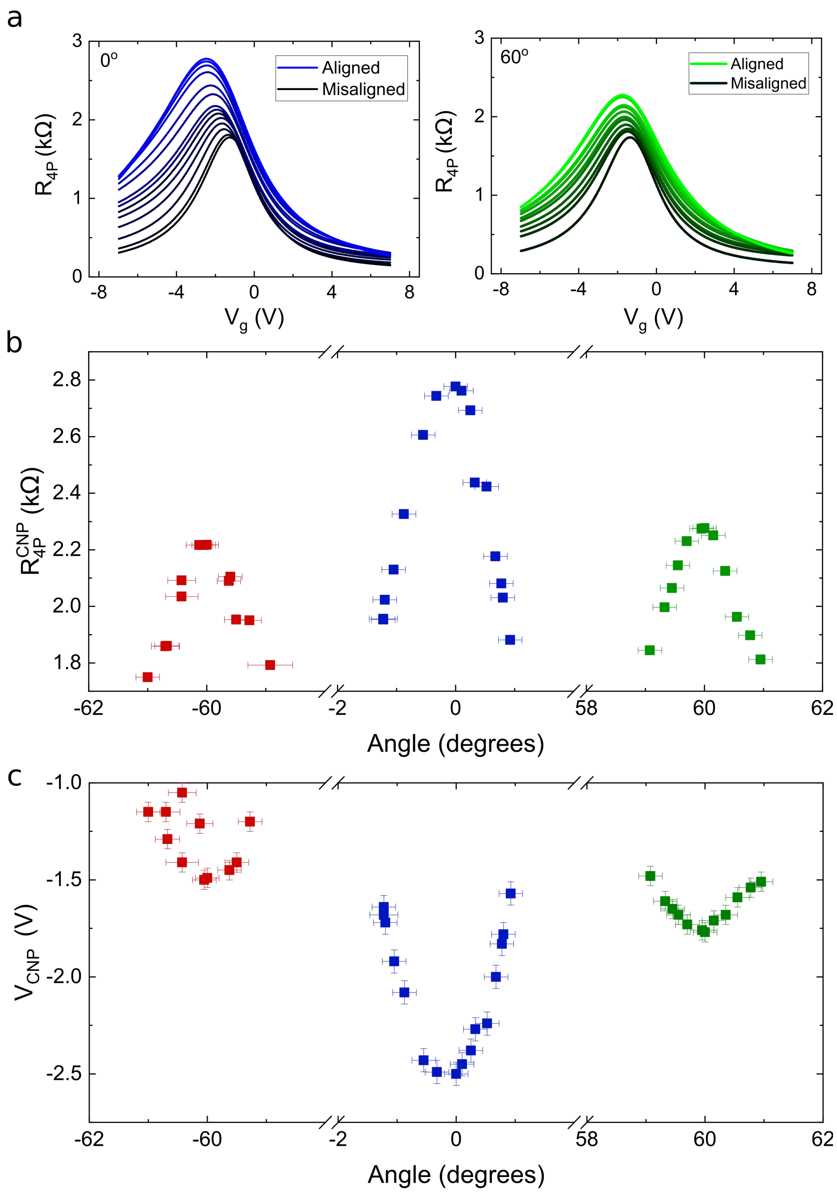

Supplementary Figure 4. Crystallographic alignment at room temperature sample II. a, Resistance as a function of the gate voltage for different crystallographic alignments around 0° and 60°. **b**, Resistance value of the CNP as a function of the angular alignment measured with the AFM around -60°, 0° and 60°. **c**, Position of the CNP as a function of the angular alignment around -60°, 0° and 60°.

The change in position of the CNP, different for 0° and 60°, reflects different levels of strain in the system. It has been demonstrated experimentally[1], and explained theoretically[2], that strain will modify the work function of graphene. This will be reflected in a change in the position of the CNP as a function of the strain. In our experiments, the existence of the commensurate state generates strain inside the moiré cell. This has the same effect in our samples, a shift on the position of the CNP in gate voltage.

Supplementary Note 3: Sample characterization

The temperature dependence of the resistance as a function of the gate voltage, Figs. 5 and 6, shows clearly the presence of satellite peaks at low temperature. Its evolution in temperature shows that the broadening of the $R(V_g)$ curves at room temperature is an indication of the appearance of the satellite peaks, hidden by thermal broadening.

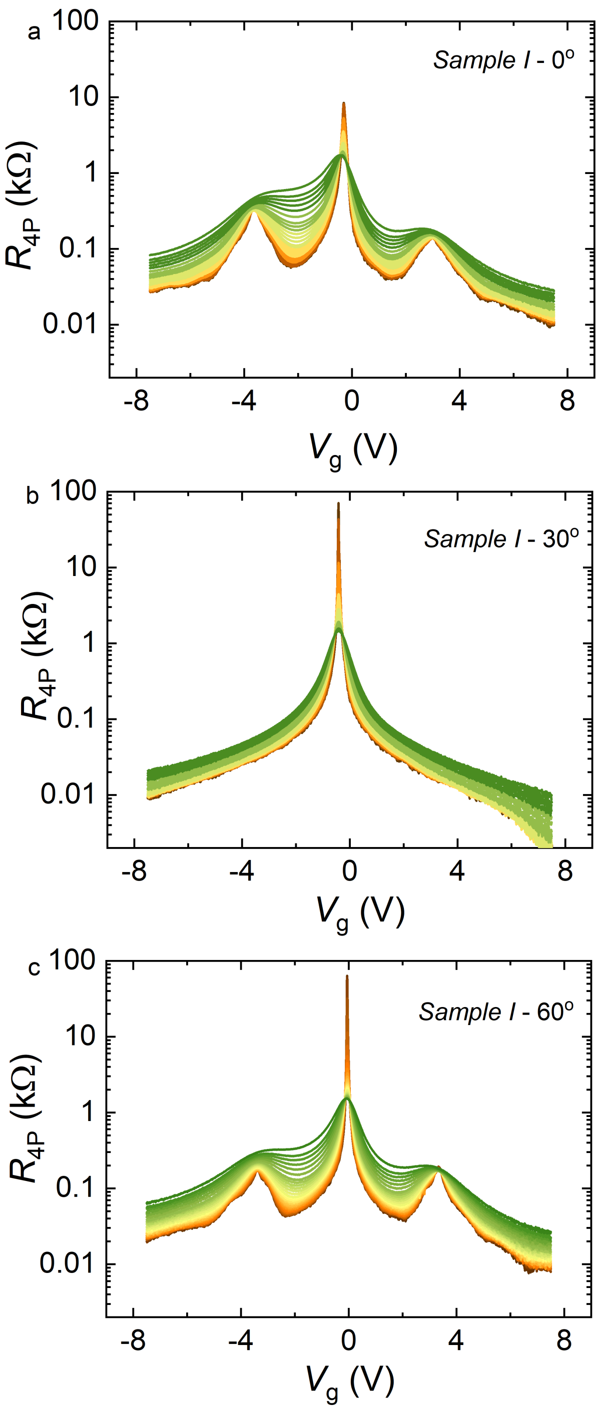

Supplementary Figure 5. Temperature dependence for the resistance of sample I. Temperature dependence of the resistance as a function of the gate voltage, in semi-Log scale for **a** 0°, **b** 30° and **c** 60° of alignment. Measurements between 1.4 K (brown) and 220 K (green).

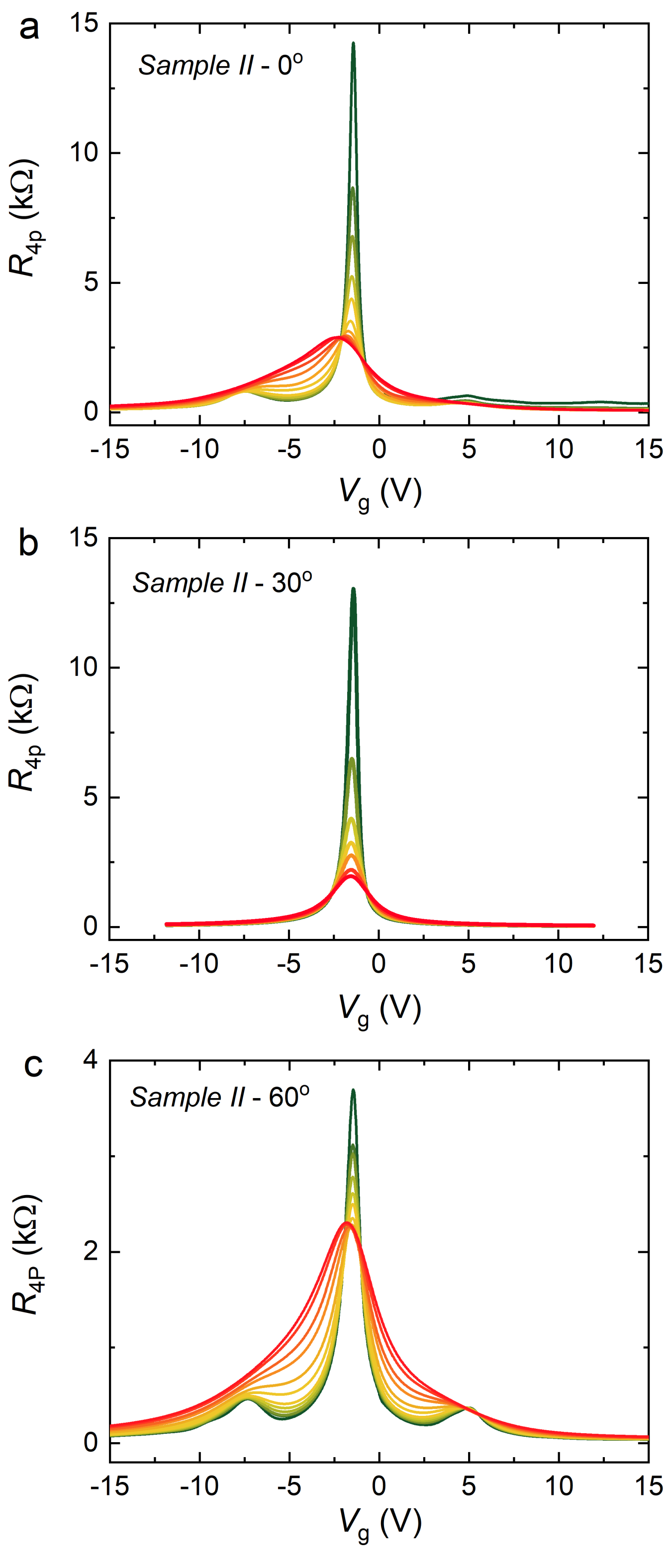

Supplementary Figure 6. **Temperature dependence sample II.** Temperature dependence of the resistance as a function of the gate voltage for **a** 0°, **b** 30° and **c** 60° of alignment. Measurements between 20 K (green) and 200 K (red).

When performing Hall resistance measurements in the presence of a low magnetic field (0.2 T), both the satellite peaks and the CNP are accompanied by sign inversion of the Hall resistance, Supplementary Figure 7, both evident at 0° and 60° alignment. At 30° case, full misalignment, there are no satellite peaks and therefore the Hall resistance stays close to zero in such regions.

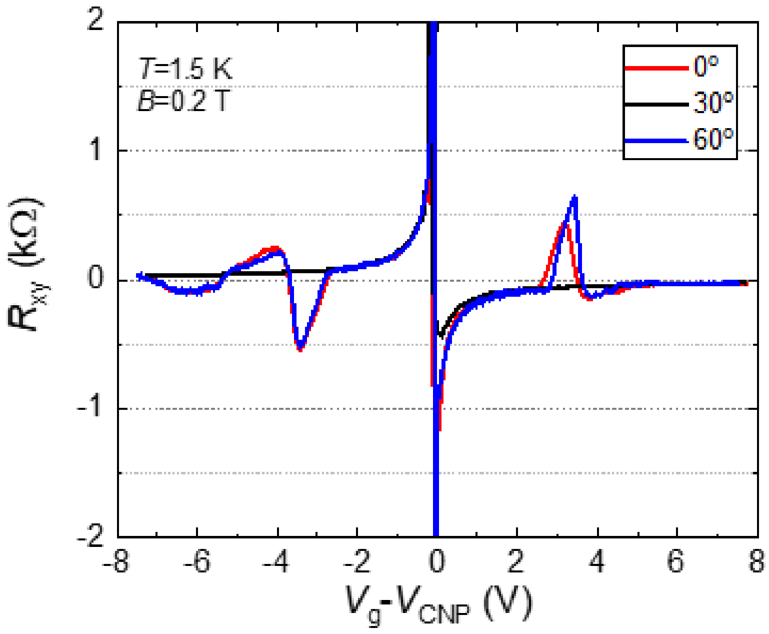

Supplementary Figure 7. Hall resistance measurement, sample I. Measurements at low temperature and 0.2 T of the Hall resistance for the three crystallographic alignments 0°, 30° and 60°.

Mean free path at different angular alignments

We calculate the mean free path versus temperature from the measurements of resistance as a function of gate voltage at different temperatures, for example Supplementary Figure 5 and 6, by using the expression:

$$l_{\mathrm{mfp}} = \frac{\sigma h}{2e^2\sqrt{\pi n}}. \tag{S1}$$

In Supplementary Figure 8 we can see that sample I is in a ballistic regime for $T < 10$ K, shadow area. We also remarked that the temperature dependence of the mean free path for the misaligned (30°) case turns out to be different for the aligned case. The understanding of this regime is out of the scope of our manuscript, since it will need to extend the measurements to higher temperatures.

From measurements of resistance as a function of the carrier density we can have an idea of the sample quality by extracting the residual carrier density, δn, from the full width at half maximum of the CNP peak. In Supplementary Figure 9 we can see that the residual carrier density for sample II is about one order of magnitude larger than for sample I. This is only a comparative measurement, unfortunately we cannot extract a quantitative value of disorder.

Also notice that this qualitative comparison refers mostly to bulk disorder and not to edge disorder.

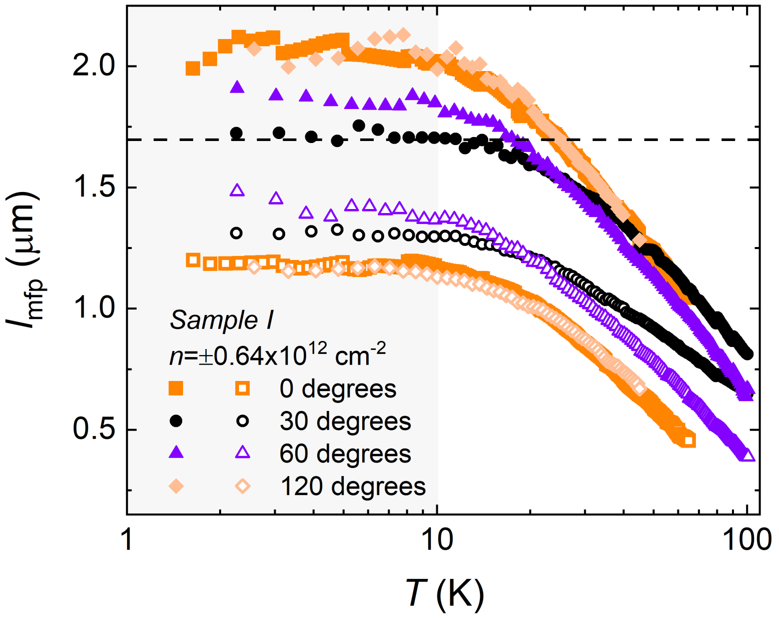

Supplementary Figure 8. Mean free path, for sample I, calculated from the resistance as a function of V_g at different temperatures for a density of $+0.64 \times 10^{12}$ cm^{-2} filled symbols and -0.64×10^{12} cm^{-2} (empty symbols) for different angular alignments. Dashed horizontal line represents the width of our sample. The shaded area corresponds to the values of temperature where the mean free path is not varying with temperature.

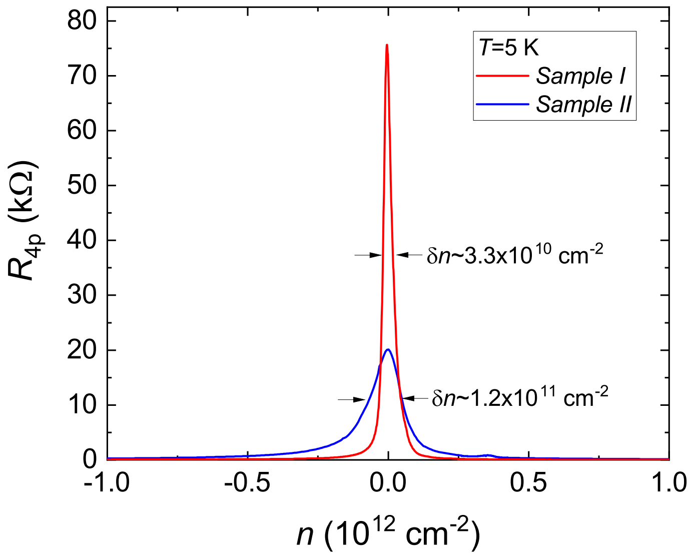

Supplementary Figure 9. Residual carrier density for sample I and II. Four probe resistance measurements as a function of the carrier density for samples I and II at $T = 5$ K in the misaligned position 30°. The full width at half maximum of the resistance peak at zero magnetic field provides a measurement of sample quality.

Supplementary Note 4: Non-local measurements

Measurement configurations

To avoid any common grounds and spurious signals we have followed the measurement scheme developed in[3]. This setup consists of an operational amplifier to keep the voltage of our sample balanced and high impedance amplifiers (CELIANS EPC-1B) to avoid any current leak, see Figure 10.

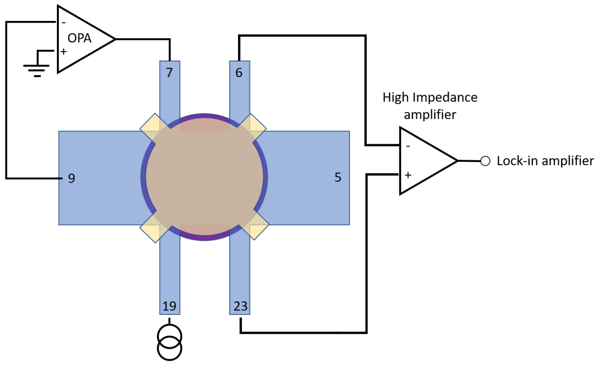

Supplementary Figure 10. Non-local measurements. Non-local configuration measurement using an operational amplifier and high input impedance voltage amplifiers.

Our non-local measurements are performed at low current (10 nA) to avoid Joule heating at the injector that could cause heat to flow into and past the detector region. The resulting temperature gradient along the detector region would give rise to a non-local voltage across the detector contacts via the Nernst effect, quantified by the transverse thermopower coefficient. This temperature gradient is proportional to the heating power, quadratic in current, and therefore contributes to the non-local voltage only at the second harmonic of the excitation frequency. As a result, Joule heating would not affect the first harmonic data presented in this report[4]

We have also tested the different configurations for both local and non-local measurements, Supplementary Figure 11. For various combinations of these we can see that the power law relation do not change.

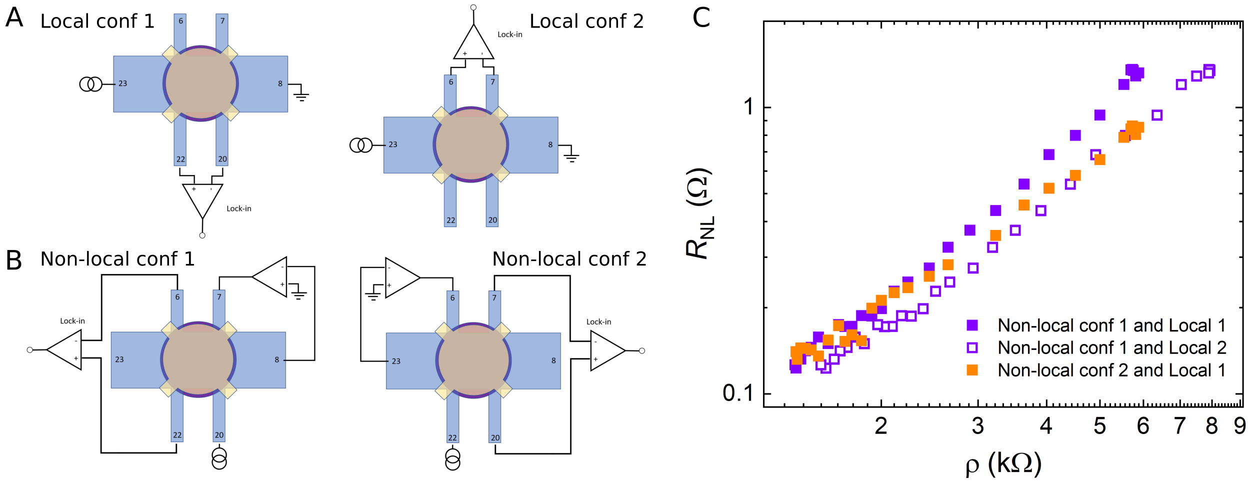

Supplementary Figure 11. Measurements configurations. **A**, local measurements configurations. **B** Non-local measurements configuration and **C** local versus non-local signal for a combination of measurements. Sample IV 60°.

Current dependence of non local measurements

To ensure that our non-local measurements are not affected by the amplitude of the current we inject, we have measured the non-local signal at different injection currents, Supplementary Figure 12a. The resemblance between the curves tells us that the magnitude of the current is not affecting our measurements. We have also performed the same measurements for different temperatures and extracted the non-local conductance (inverse of the non-local resistance at the CNP) as a function of the

temperature for 1 nA, 10 nA and 100 nA, Supplementary Figure 12b. We can see that at low temperatures for the highest measured current (100 nA) there is a slight effect reflected by a small increase of the non-local conductivity.

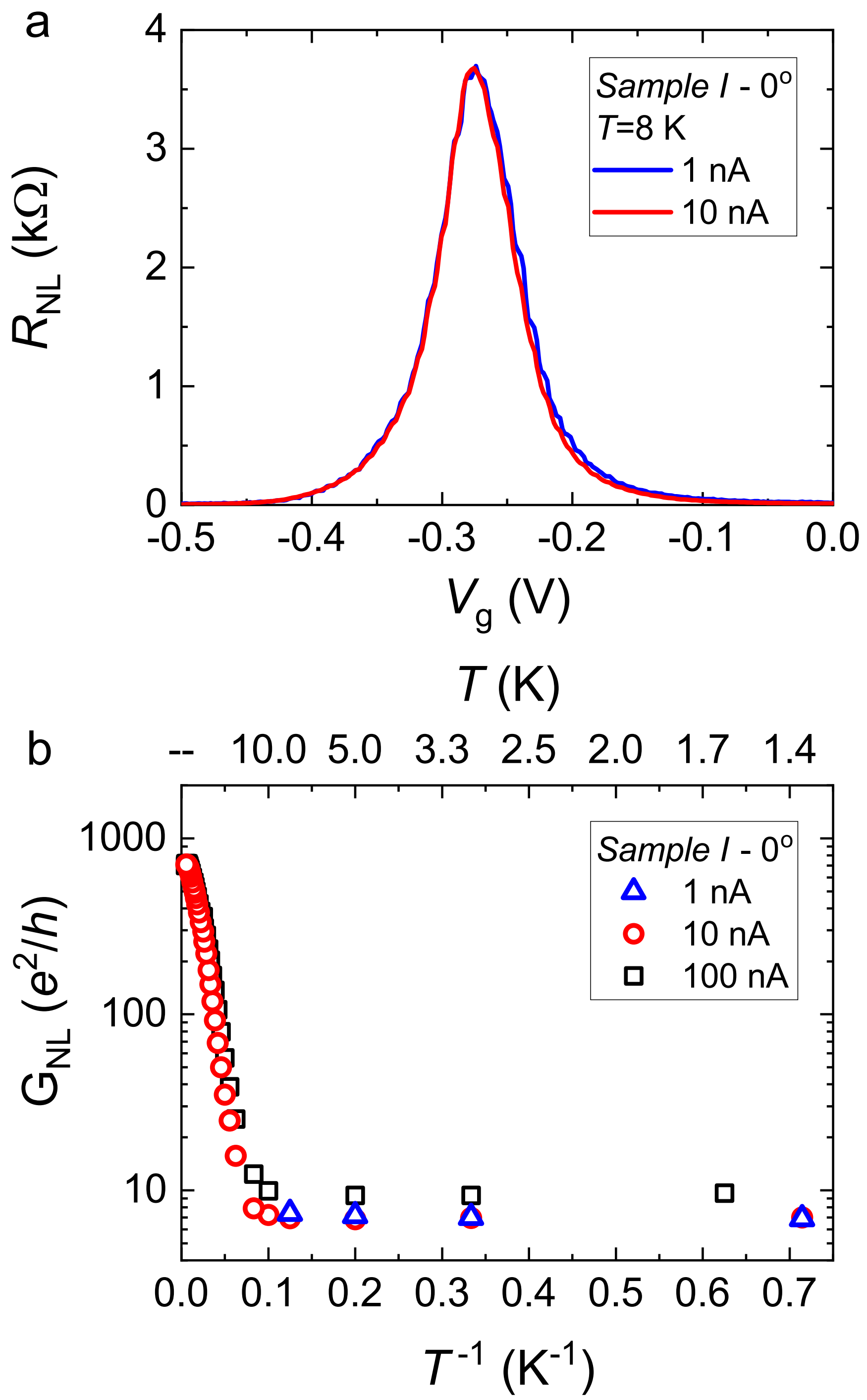

Supplementary Figure 12. Non-local measurements for different applied current, sample I. **a**, non-local resistance as a function of the gate voltage for 1 nA and 10 nA. **b**, Non-local conductivity as a function $1/T$ for 1 nA, 10 nA and 100 nA.

Supplementary Note 5: Determining the moiré wavelength by magneto transport measurements

By using magneto transport measurements we can determine the magnetic field at which one flux quantum threads the supperlattice unit cell. To obtain this we plot the longitudinal resistance as a function of the gate voltage (or carrier density) and $1/B$, Supplementary Figure 13. In this plot we can see the appearance of equally spaced horizontal lines that appear when the Landau fans coming from the charge neutrality point and the satellite peaks intercept each other. It is important to remark that this is a purely geometrical effect and gives therefore a very accurate estimation of the moiré superlattice size[5].

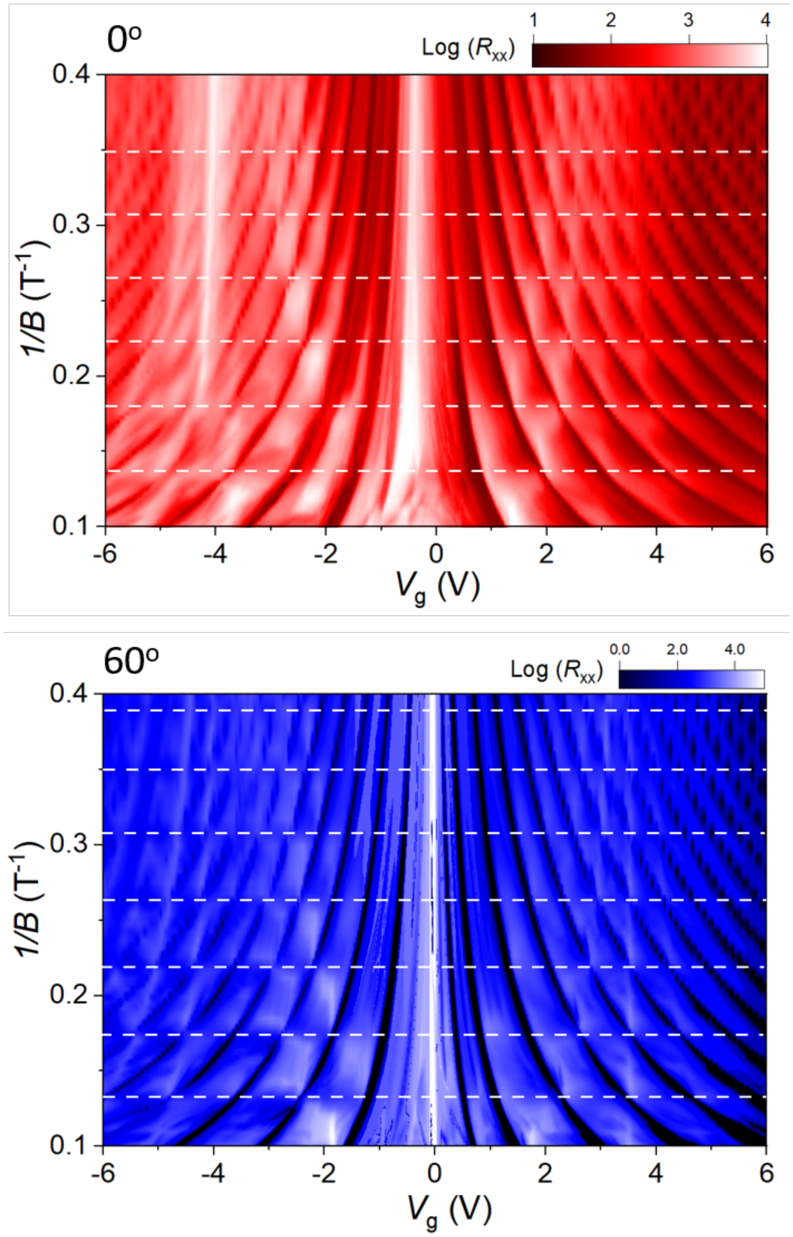

Supplementary Figure 13. **Magneto transport measurements, sample I**. Longitudinal resistance, in log scale, as a function of the applied gate voltage and the inverse magnetic field for **a** 0° and **b** 60° of alignment at 1.4 K. Horizontal dashed lines are guides for the eyes.

We obtain a space between these horizontal lines corresponding to $B_0 = 24.16$ T and $B_0 = 22.69$ T. This corresponds to λ=14.1±0.4 nm and λ=14.5±0.3 nm for 0° and 60°, respectively.

Supplementary Note 6: Temperature dependence and thermally activated regimes for the local and non-local resistance

In our local measurements we observe different transport regimes at the CNP: first a thermally activated regime, fit with an Arrhenius law, and a hopping regime for temperatures lower than 10 K. In Supplementary Figure 14, we plot an example for the activation regime of the CNP for the measurements at 0°, 30°, 60° and 120° in local (a) and non-local (b) configuration, sample I. The values for the local and non-local energy gaps at the CNP for different alignments are summarized in the main text and in Supplementary Figure 14c.

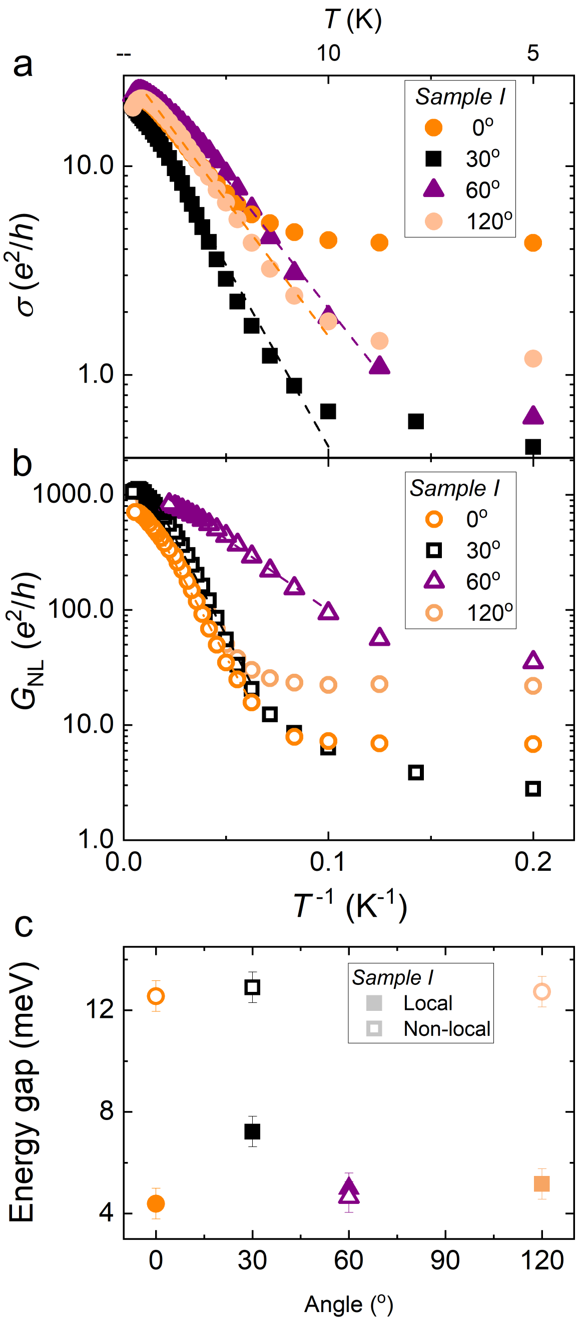

Supplementary Figure 14. Temperature dependence of the local and non-local measurements at the CNP for 0°, 30° **and** 60°**, sample I. a** and **b**, Arrhenius plot for the energy gap at the CNP for the local and non-local measurements, respectively. **c** Energy gaps for the sample described in the main text.

If we compare the energy gap obtained from the local and non-local measurements, for the 0° and 120° measurements, we can easily see that, as expected, there is approximately a factor of three of difference between these energy gaps, in agreement with[6,7], and supporting of the cubic relation between the local and non-local signals. It is also important to notice that the extracted values for the energy gaps, local and non-local, are at least a factor of two larger than previously reported[7], reaffirming the high quality of our samples.

In contrast, we do not observe any activation regime at the satellite peaks , Supplementary Figure 15, for sample I nor sample II Supplementary Figure 16. However, we do observe a change in behavior in the electron side but for opposite alignments (60° in sample I and 0° for sample II). This behavior remains to be investigated and could be associated to the different intrinsic displacement fields in the samples.

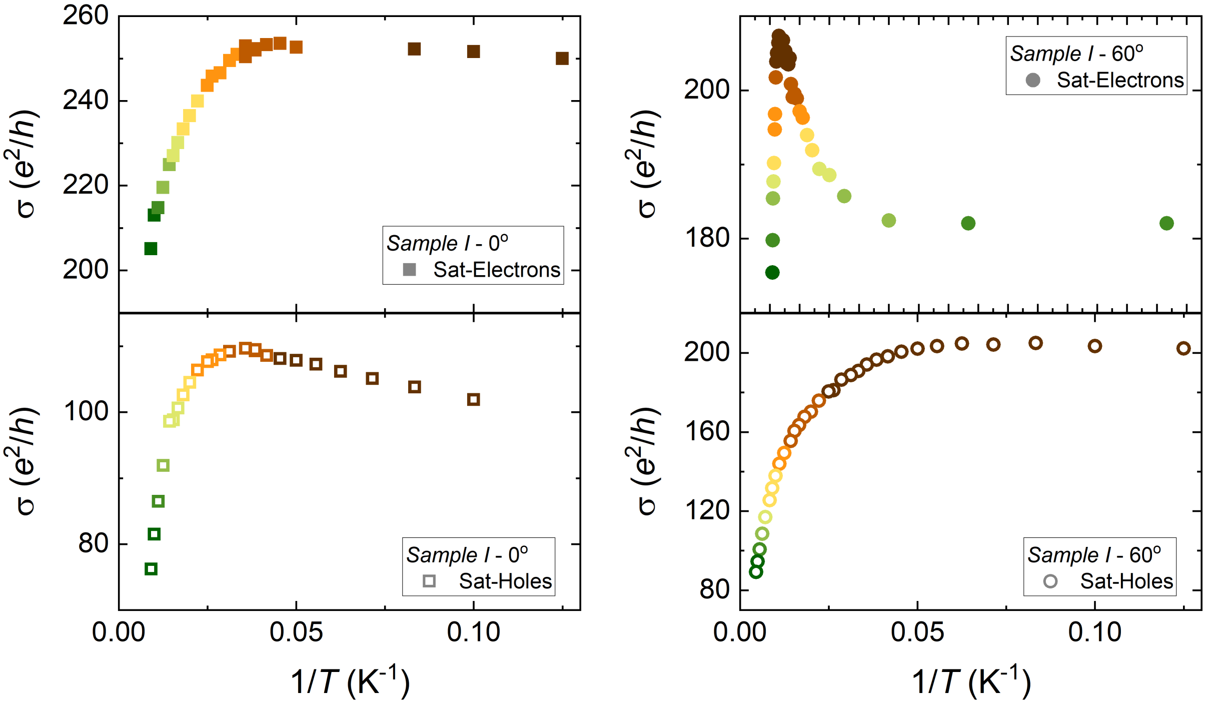

Supplementary Figure 15. Temperature dependence local measurements at the satellite peaks, sample I. Measurements at the satellite peak, for 0° (**top**) and 60° (**bottom**), for negative (electrons) and positive (holes) values of the gate voltage applied to the graphite gate, while the Si gate is kept at high doping.

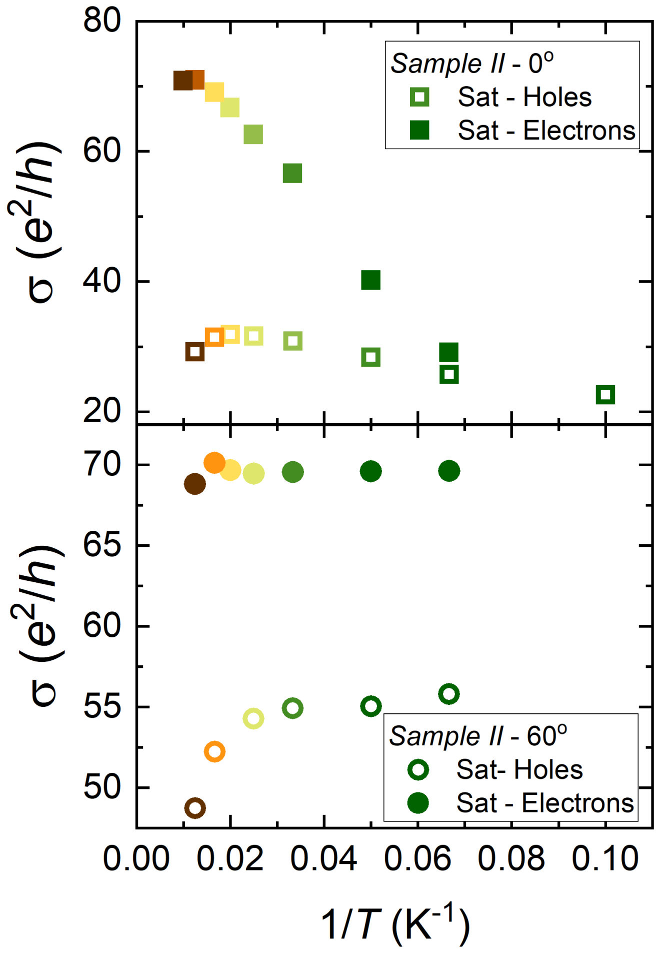

Supplementary Figure 16. Temperature dependence local measurements at the satellite peaks, sample II. Measurements at the satellite peak, for 0° (**top**) and 60° (**bottom**), for negative (electrons) and positive (holes) values of the voltage applied to the graphite gate.

Supplementary note 7: Valley Hall effect, complementary measurements

Valley Hall effect around the charge neutrality point

The cubic relation of the non-local resistance as a function of the local resistance can be observed inside the conduction and valence band also. In Supplementary Figure 17 we show the same cubic relation for values of gate voltage around the CNP.

It is important to remark that the cubic relation is also observed outside of the disorder window δ_n, obtained in supplementary note 3, this can be seen in the insert of supplementary figure 17 we delimited the value of δ_n in a grey area. We can see that for measurements outside this region (marked by green dashed lines) we can still observe the cubic dependence revealing the valley Hall effect.

Measurements at 120 degrees

We have performed local versus non-local measurements for 120° alignment, sample I. However, at the moment of the measurements contact #6 was broken, Supplementary Figure18a. This clearly impacted the saturation regime of our measurements, since it changes the geometrical configuration of our sample. However, a nearly cubic relation, $\rho^{2.5}$ is still visible, Supplementary Figure 18b. Considering that the width of our sample is divided by a factor of two given the change in configuration, and using equation (2) of the main text, the expected value for the non-local resistance in the fully developed valley Hall regime is ≈ 1.8 kΩ (dashed line in Supplementary Figure 18b), instead we obtain ≈ 1.2 kΩ, which we consider in good agreement.

It is also important to remark that, in the same fashion as for the measurements at 0°, for temperatures higher than 70 K the $\rho^{2.5}$ dependence is lost.

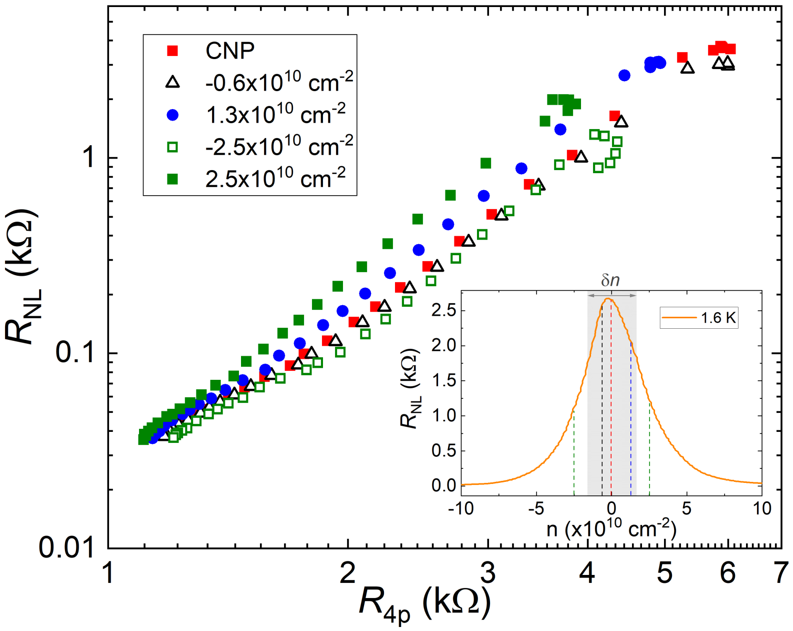

Supplementary Figure 17. **Cubic relation around the CNP for 0°, sample I**. The same cubic relation can be observed for values of densities close to the CNP. Insert: non-local measurement for 0° at 1.6 K, gray area represents the disorder dominated regime extracted in Supplementary Figure 9, the color of the dashed lines correspond to the different densities marked in the legend.

Angular dependence in other samples

For Sample II, the global graphite gate generates a very large contact resistance when passing through the CNP, since there is a part of our graphene flake that is exposed and therefore not as clean as the part covered by the BN handles. This sample architecture was improved by the ones presented in the main text (sample I and III).

In sample II (and IV) we can still observe a nearly cubic relation of the non-local and local resistance, $R_{\mathrm{NL}} \propto \rho^{2.7}$ ($R_{\mathrm{NL}} \propto \rho^{2.8}$). This relation is strongly modified for 30° and 60° of alignment, Supplementary Figure 19 (Supplementary Figure 20). It is important to notice that technical problems prevent us from using the Si gate of Sample IV and therefore the tuning of the contacts, gated by the Si gate, is not performed in this measurements. However, we can see in Supplementary Figure 20 that the local-non-local relation is reproducible in a full 180° rotation (from -60° to 120°). It is important to keep in mind that the difference in absolute value of the non-local and local resistance are due to a ”cleaning” effect of the rotatable BN layer. Besides this change of absolute value the relation between the local and non-local remain resistance remains the same after 180° rotation.

It is important to point out that measurements on sample II were performed in a dry 4 K cryostat with a resistive coil with max ±300 mT. As we should expect the resistive coil to has no residual field, the non-local signal cannot be attributed to remnant field in the magnet.

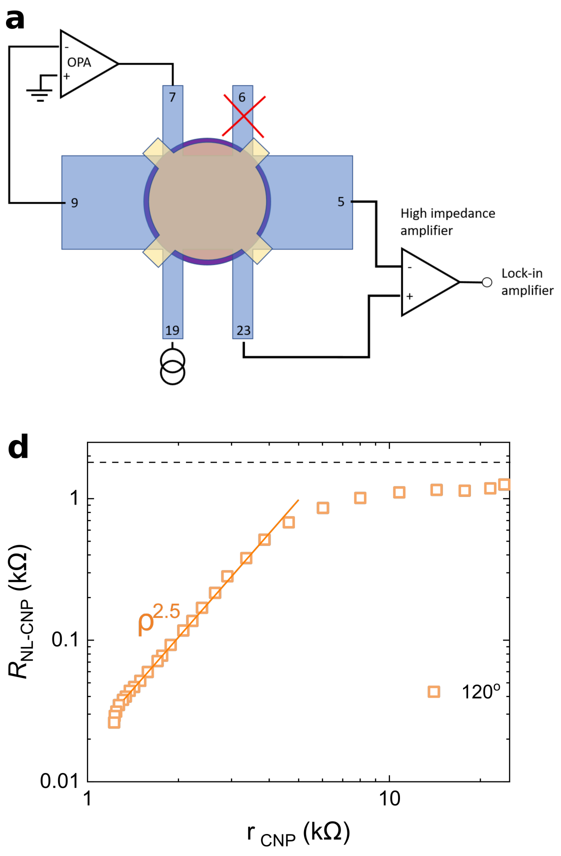

Supplementary Figure 18. Non-local resistance versus local resistivity at 120° of alignment in sample I. **a**, Measurements configuration after one contact got broken. **b**, Non-local resistance versus local resistivity for 120° of alignment, sample I. Measurements between 4 K and 110 K.

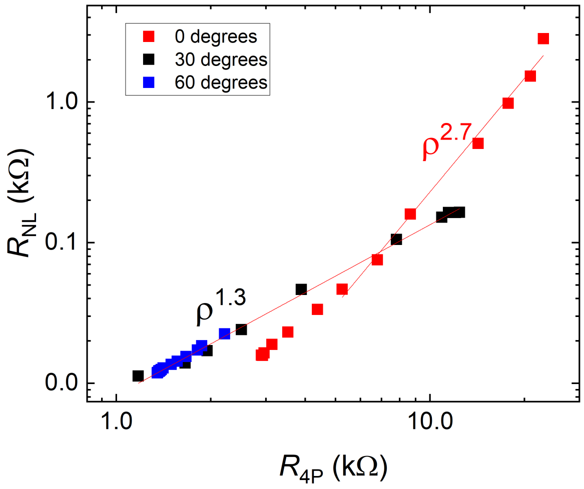

Supplementary Figure 19. Non-local versus local resistance for sample II. Non-local resistance versus four probes resistance at 0°, 30° and 60°. Temperature range between 8 K and 150 K.

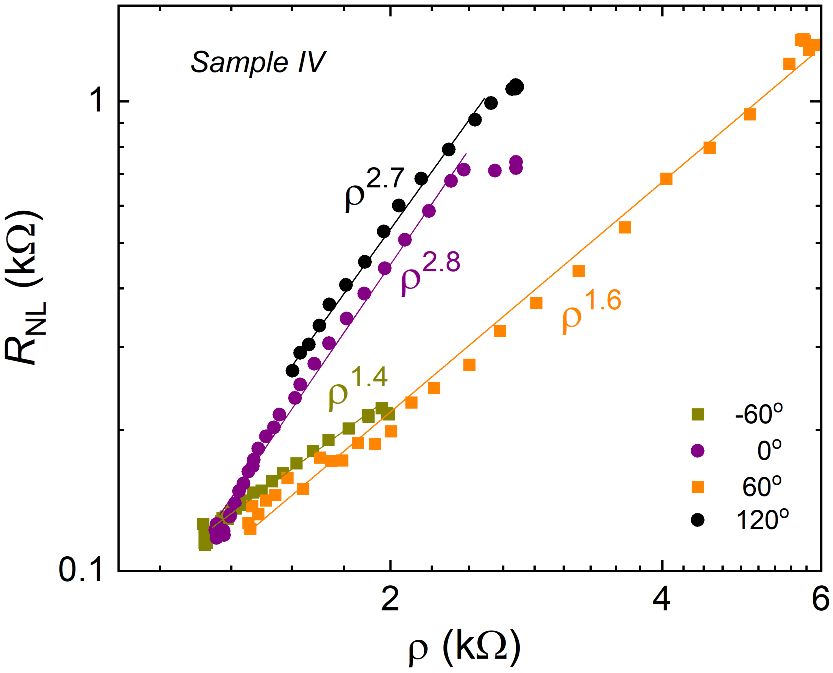

Supplementary Figure 20. Non-local versus local resistance for sample IV. Non-local resistance versus four probes resistance at -60°, 0°, 60° and 120°. Temperature range between 1.4 K and 90 K.

Supplementary note 8: Non-local signal in magnetic field

Non-local measurements as a function of gate voltage and magnetic field are similar to a transverse magnetic focusing (TMF) measurement but in a symmetric configuration. This means that the observed focusing lines are a reflection of the matching of cyclotron orbits with the distance between electrodes. This measurements are highly sensitive to modifications on the band structure and can reflect for example the presence of van Hove singularities at saddle points in the band structure[8]. Although the detailed analysis of these 2D plots is out of the scope of this manuscript, we would like to point out the main differences in these 2D plots, which reflect differences in the electronic band structure of the different alignments[8,9].

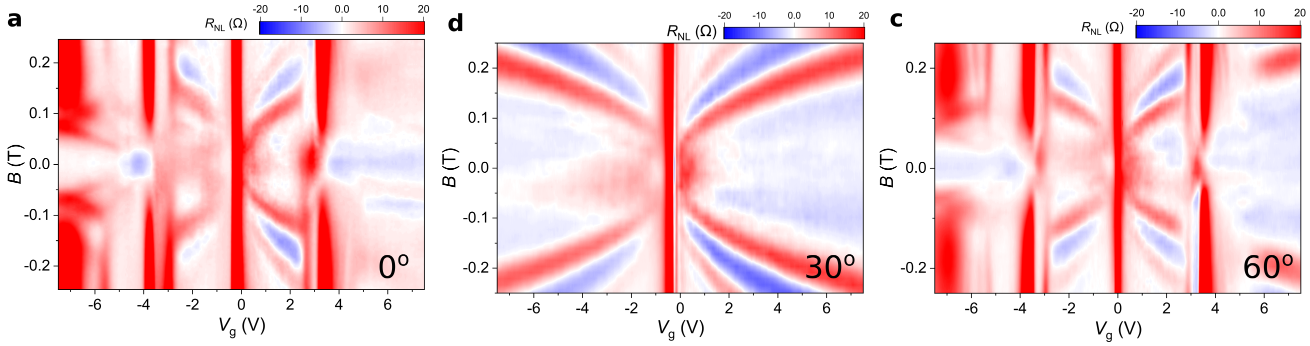

Supplementary Figure 21. Non-local resistance as a function of the magnetic field and density, sample I. a, 0°; **b**, 30° and **c**, 60°. All measurements are taken at 10 K.

The strong differences in the non-local response resembles the one of systems with very different electronic band structures, as we proposed in the main text. In Supplementary Figure 21 we can observe the TMF 2D plots for three crystallographic alignments: 0°, 30° and 60°. We highlight the main features that support the hypothesis of strongly different electronic band structures. The 2D map observed at 30°, Supplementary Figure21b, is the expected one for the unaltered band structure of

bilayer graphene[10]. In this we observe uninterrupted magneto focusing lines through all the carrier density range. In the case of 0° and 60° we observe an increase of the resistance at the gate voltage which corresponds to the satellite peak. This has been attributed to the presence of saddle points in the band structure[8]. Between the two satellite peaks the TMF signal do not show any particular behaviour. However, for carrier densities beyond the satellite peaks we observe strong differences between the two alignments, the most remarkable one being the fact that the magnetic focusing peaks of the central band seem to propagate beyond the satellite peak in the 60° of alignment, Supplementary Figure 21c. The strong difference between these TMF plots is a clear indication of the existence of different electronic band structures.

Supplementary note 9: Stacking configurations for BN/bilayer graphene

In Supplementary Figure 22 we show all the possible atomic configurations, from the more energetically favorable[11] BA to the least one AA. For all of those we can see that a sixty degrees rotation of the BN layer gives rise to a different atomic configuration.

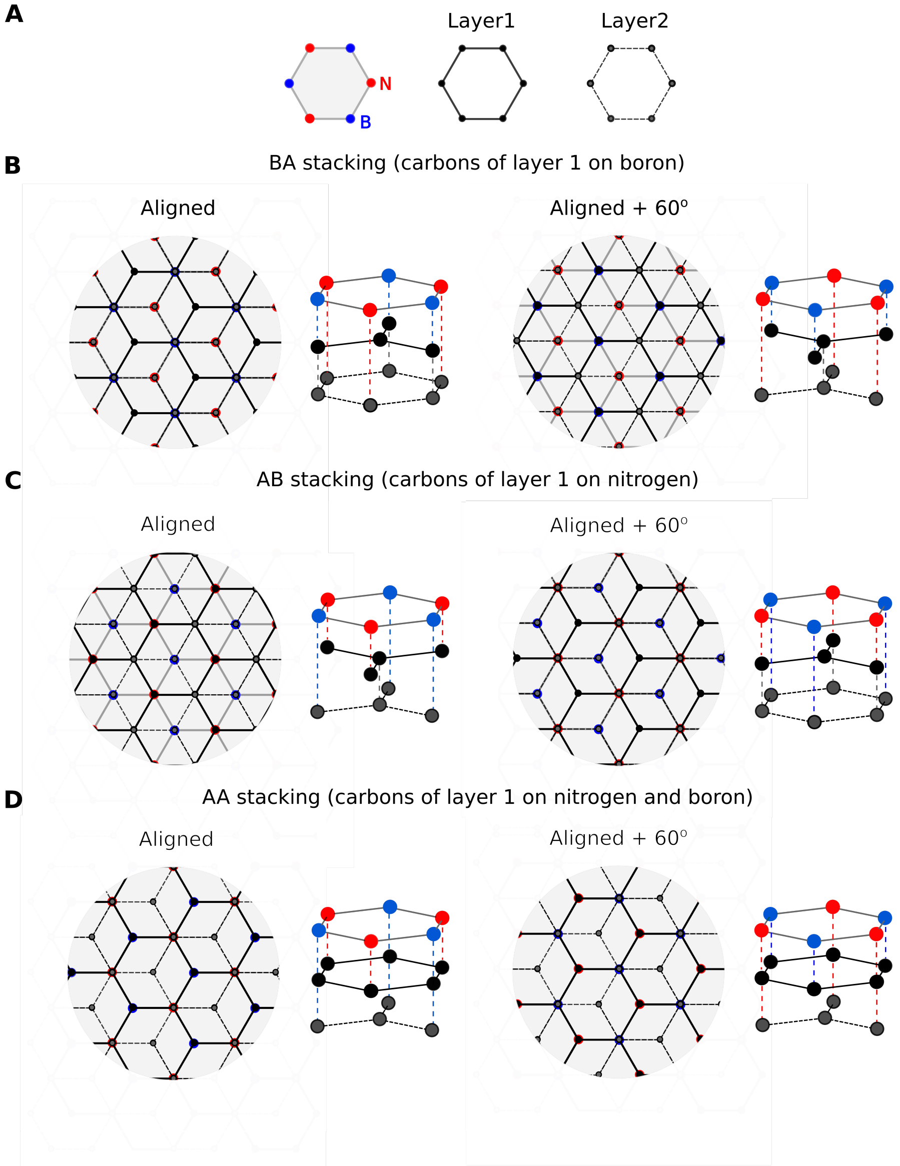

Supplementary Figure 22. Stacking configurations for BN/bilayer graphene. **A**, decription of the differnet layers. **B, C and D**, Atomic configurations inside the moiré cell, in the commensurate state, for different stacking configurations of the first layer with respect to the BN: BA - carbon on boron; AB - carbon on nitrogen and AA - carbon in boron and nitrogen. Each is represented for both aligned positions 0° and 60°.

Supplementary note 10: Sample for structural characterization

We have prepared a sample to measure with the AFM the out of plane atomic displacement, wrinkles around the moiré cell are formed as a consequence of the atomic in plane displacement inside the moiré cell. This sample consist of BN and bilayer graphene on surface. The two crystals have been pre-aligned at the moment of the preparation by aligning their crystallographic edges. A picture of this sample and its cross section are shown in Supplementary Figure 23.

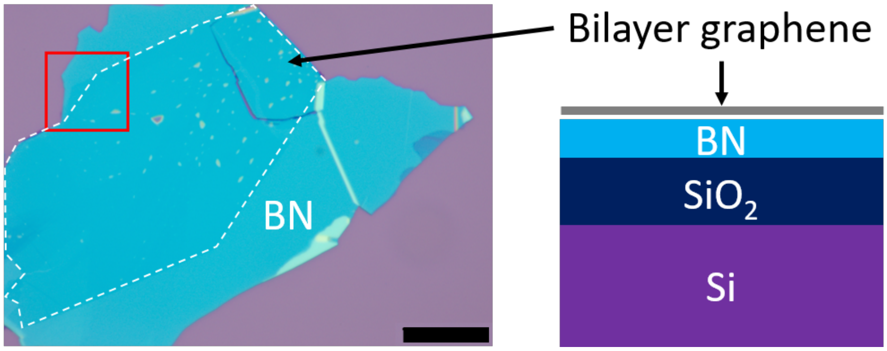

Supplementary Figure 23. Sample for structural characterization. Optical micrograph of the sample (left). Dashed line represents the graphene area. Scale bar 20 μm. Cross section of the sample (right).

The bilayer character of the sample has been tested by Raman spectroscopy. The AFM measurement in this sample, shown in the main text shows the clear presence of a moiré superlattice where the in-plane atomic displacement gives rise to relaxation regions around the moire cell.

Supplementary note 11: Electronic models

To compute the electronic structure of the considered graphene/hBN systems, we employed the p_z tight-binding Hamiltonian, similar to those presented in[12,13]. In particular, the Hamiltonian is written as

$$H_{tb} = \sum_n V_n a_n^\dagger a_n + \sum_{n,m} t_{nm} a_n^\dagger a_m$$

where the on-site energies V_n = 0, 3.34 eV, and -1.4 eV for carbon, boron, and nitride atoms, respectively. The hopping energies t_{nm} are determined using the standard Slater-Koster formula

$$\begin{aligned} t_{nm}(r_{nm}) &= V_{pp\pi} \sin^2\phi_{nm} + V_{pp\sigma}\cos^2\phi_{nm}, \\ V_{pp\pi} &= V_{pp\pi}^0 \exp\left((a_0 - r_{nm})/r_0\right), \\ V_{pp\sigma} &= V_{pp\sigma}^0 \exp\left((d_0 - r_{nm})/r_0\right) \end{aligned}$$

where the direction cosine of $\vec{r}_{nm}$ along Oz axis is $\cos\phi_{nm} = z_{nm}/r_{nm}$, $r_0 = 0.184a$, $a_0 = a/\sqrt{3}$, and $d_0 = 3.35$Å while $a \simeq 2.49$Å.

Atomic structure relaxation and electronic band structures

More details related to atomic structure relaxation are presented in Supplementary Figure 24, including additionally the variation of interlayer distance between graphene/hBN layers and the modification of stacking structure, that is due to atomic reconstruction effects. In Supplementary Figure 25, the electronic band structures computed for both unrelaxed and relaxed lattices are displayed. While it is almost negligible in the unrelaxed case, the presented results clearly demonstrate that the effects of atomic structure relaxation essentially govern the significant difference in the electronic structures of 0°- and 60°-alignments.

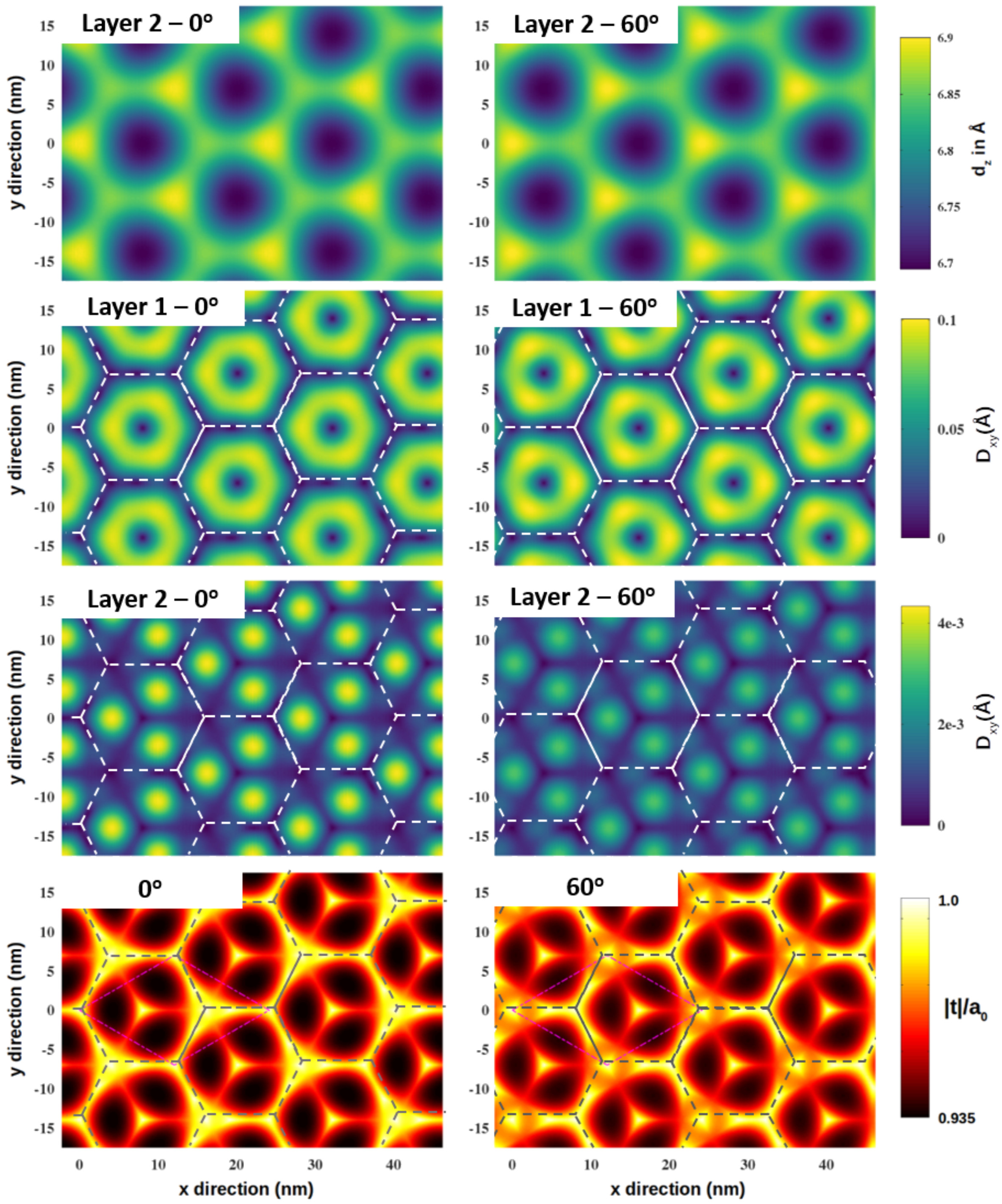

Supplementary Figure 24. Atomic structure relaxation. Bilayer graphene/hBN are aligned at 0° (left) and 60° (right). The variation of interlayer distance d_z between the second graphene layer and hBN one is presented in the two top images. The four images below illustrate the variations of the in-plane displacements D_{xy} in both graphene layers. At last, the two bottom images display the modification of stacking configuration between two graphene layers, by illustrating the variation of stacking vector **t**. **t** is determined as the translation vector applied locally to a C-ring of one graphene layer to recover the AA stacking configuration at the considered position, i.e., $|\mathbf{t}| = a_0$ and 0 for the local AB and AA stacking configurations, respectively.

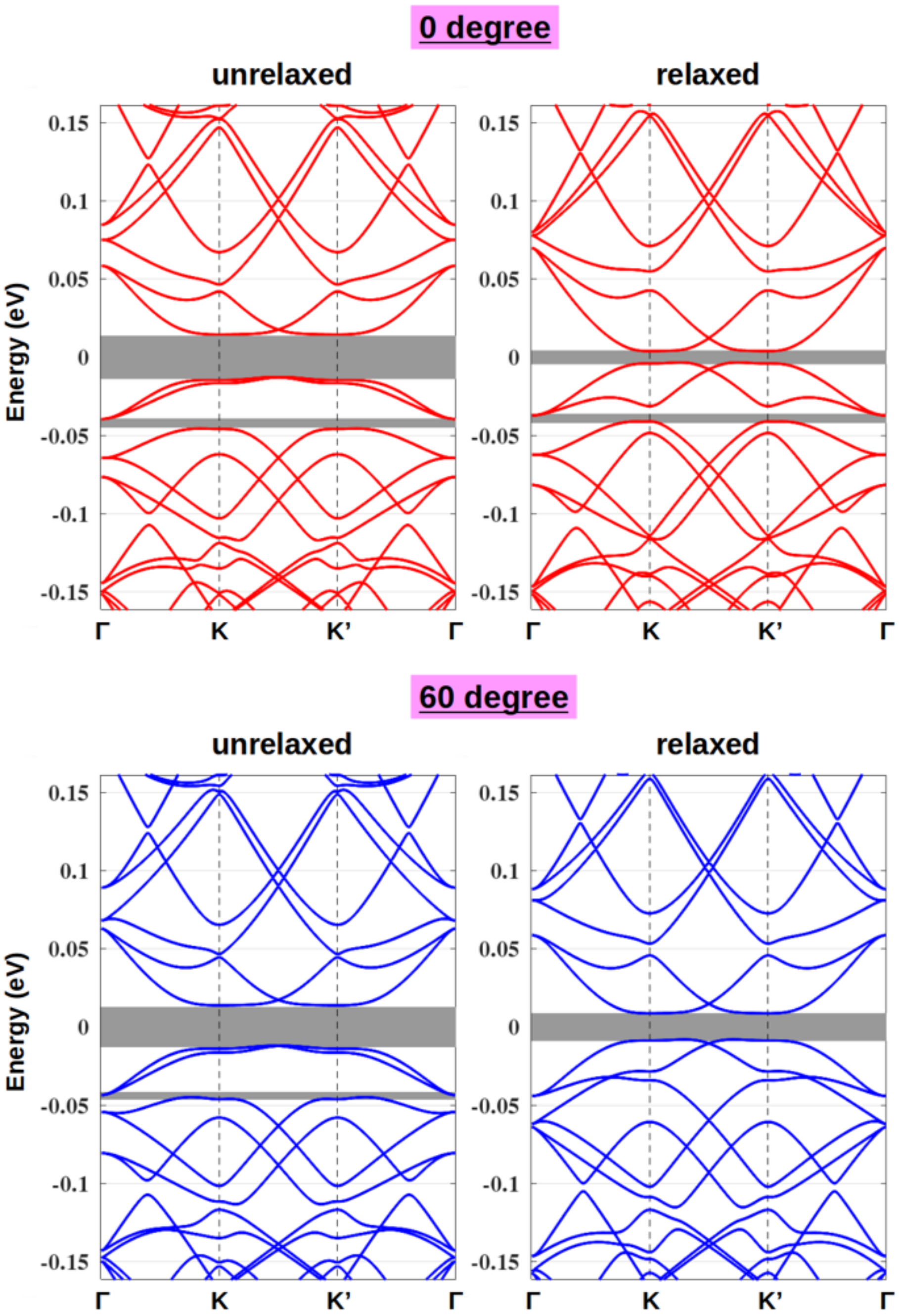

Supplementary Figure 25. Electronic band structures. Bilayer graphene/hBN are aligned at 0° (top) and 60° (bottom). Ideal pristine atomic structure of the graphene layers are preserved on the left, while atomic structure relaxations are considered on the right. Numerical simulation without the presence of displacement field.

Supplementary note 11: Other theories applied to the valley Hall effect

The valley Hall effect can also be interpreted in terms of two leading theories:

Berry curvature driven valley currents: If we consider the existence of a Berry curvature, $\Omega(\mathbf{k})$[3, 14–17], which acts as a pseudo-magnetic field, this will give rise to an "anomalous" velocity, perpendicular to the external longitudinal electric field $\mathbf{E}$. The electron velocity, $\mathbf{v}$, then becomes:

$$\mathbf{v}(\mathbf{k}) = \frac{\partial \varepsilon(\mathbf{k})}{\hbar \partial \mathbf{k}} - \frac{q}{\hbar}\mathbf{E} \times \Omega(\mathbf{k}), \qquad \text{(S2)}$$

where k is the wavevector, $\varepsilon(\mathbf{k})$ is the band energy, q is the carrier charge and $\hbar$ is the reduced Planck constant. From equation S2 we can see that the larger the Berry curvature, the stronger will be the anomalous velocity. The Berry curvature in bilayer graphene is given by[18]:

$$\Omega(\mathbf{p}) = \frac{2\hbar^2 v^4 \gamma \Delta |\mathbf{p}|^2}{(v^4 \mathbf{p}^4 + \gamma^2 \Delta^2)^{3/2}}, \tag{S3}$$

where Δ is the value of the energy gap, γ is the interlayer coupling, v is the Fermi velocity in monolayer and $\mathbf{p}$ is momentum. This means that the Berry curvature will spike to a maximum at very small values of the energy gap and then reduce rapidly as the energy gap increases. We could therefore explain the contrast between 0° and 60° by a difference in the energy gaps at the CNP as predicted by our numerical simulations without displacement field, Fig 25. However, this is not consistent with our experimental observations where the energy gap is, between error bars, the same for all the alignments. In the following section we present calculations of the Berry curvature from the electronic band structures presented in the main text.

Spatially varying regions of broken sublattice symmetry: Recent theoretical calculations propose that the valley Hall effect observed in monolayer graphene aligned with BN[14,15,17] originates from the spatial variation of the broken sublattice symmetry[19]. If this effect is at the origin of the valley Hall effect in monolayer graphene the picture becomes more complicated when dealing with bilayer graphene. Following the results of our numerical simulations we can say that the spatial variations of broken sublattice symmetry will be different between the two layers, and it will always exist for the first layer. It is then not evident why the valley effect is observed for only one of the two layer alignments, and clearly, further numerical investigations would be needed to clarify the situation.

Supplementary Note 12: Berry curvature calculation

The Berry curvature is numerically computed using the following equation[20]

$$\Omega_n(\mathbf{k}) = \hbar^2 \sum_{m \neq n} \frac{-2\mathrm{Im}[\langle n\mathbf{k} | \hat{v}_x | m\mathbf{k} \rangle \langle m\mathbf{k} | \hat{v}_y | n\mathbf{k} \rangle]}{(\varepsilon_{n\mathbf{k}} - \varepsilon_{m\mathbf{k}})^2} \tag{S4}$$

where $|n\mathbf{k}\rangle$ and $\varepsilon_{n\mathbf{k}}$ are the eigenwavefunctions and eigenvalues, respectively, computed using the tight binding Hamiltonian presented above. The numerical resutls for each alignment are presented in Supplementary Figure 26 for both alignments.

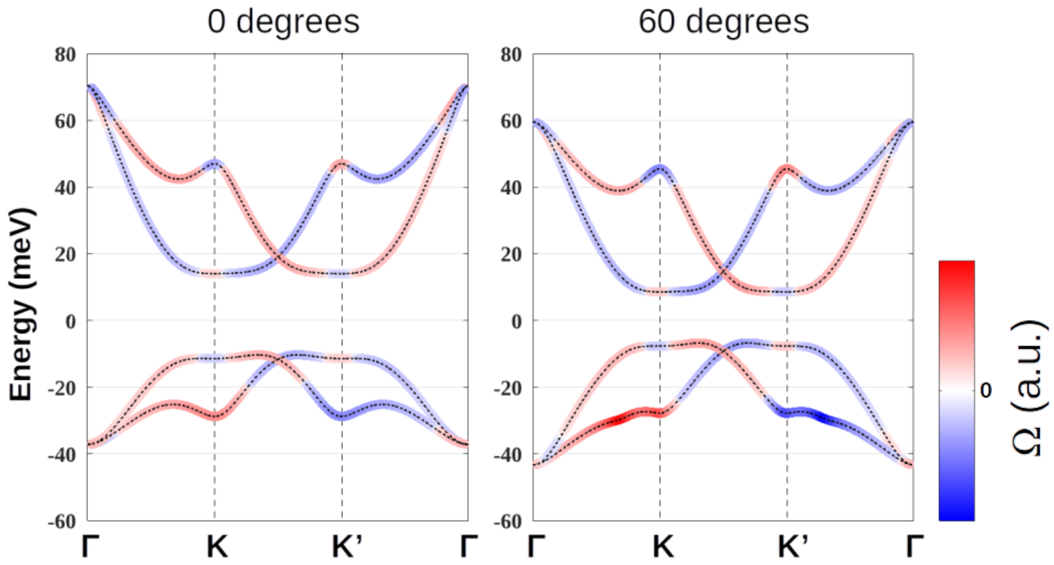

Supplementary Figure 26. Berry curvature computed near the zero energy point of fully aligned structures and when a displacement field of -0.1 V/nm is applied (see Supplementary Figure4 in the main text).

Supplementary note 13: Effective mass

Given their complexity as seen in Supplementary Figure4, the low energy bands of two 0° and 60° aligned cases can not be described using a simple effective model. Hence, these bands are zoomed in and presented in Supplementary Figure 27 and by this way, their effective masses can be estimated and roughly compared. In particular, by considering the band curvature, we can conclude that the charge carriers in the 0° case are heavier than those in the 60° one.

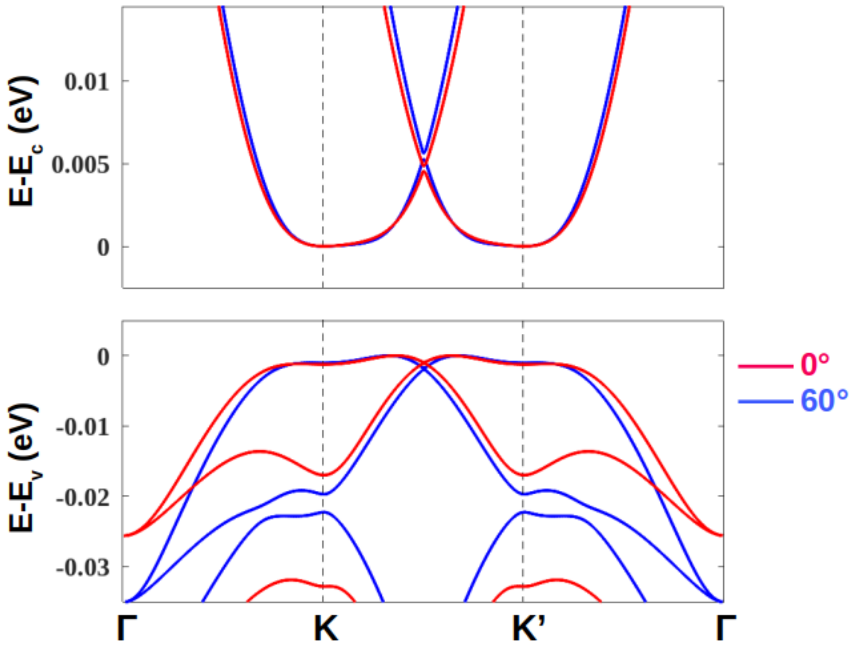

Supplementary Figure 27. Effective mass comparison: the bandstructures of both aligned cases in Supplementary Figure4 are zoomed in around the bandgap.

Supplementary References

1. Wang, L. *et al.* Global strain-induced scalar potential in graphene devices. *Commun. Phys.* **4**, 1–6, DOI: 10.1038/s42005-021-00651-y (2021).
2. Choi, S.-M., Jhi, S.-H. & Son, Y.-W. Effects of strain on electronic properties of graphene. *Phys. Rev. B* **81**, 081407, DOI: 10.1103/PhysRevB.81.081407 (2010).
3. Shimazaki, Y. *et al.* Generation and detection of pure valley current by electrically induced Berry curvature in bilayer graphene - Nature Physics. *Nat. Phys.* **11**, 1032–1036, DOI: 10.1038/nphys3551 (2015).
4. Renard, J., Studer, M. & Folk, J. A. Origins of Nonlocality Near the Neutrality Point in Graphene. *Phys. Rev. Lett.* **112**, 116601, DOI: 10.1103/PhysRevLett.112.116601 (2014).
5. Hunt, B. *et al.* Massive Dirac Fermions and Hofstadter Butterfly in a van der Waals Heterostructure. *Science* **340**, 1427–1430, DOI: 10.1126/science.1237240 (2013).
6. Yamamoto, M., Shimazaki, Y., Borzenets, I. V. & Tarucha, S. Valley Hall Effect in Two-Dimensional Hexagonal Lattices. *J. Phys. Soc. Jpn.* **84**, 121006, DOI: 10.7566/JPSJ.84.121006 (2015).
7. Endo, K. *et al.* Topological valley currents in bilayer graphene/hexagonal boron nitride superlattices. *Appl. Phys. Lett.* **114**, 243105, DOI: 10.1063/1.5094456 (2019).
8. Lee, M. *et al.* Ballistic miniband conduction in a graphene superlattice. *Science* (2016).
9. Berdyugin, A. I. *et al.* Minibands in twisted bilayer graphene probed by magnetic focusing. *Sci. Adv.* **6**, eaay7838, DOI: 10.1126/sciadv.aay7838 (2020).
10. Taychatanapat, T., Watanabe, K., Taniguchi, T. & Jarillo-Herrero, P. Electrically tunable transverse magnetic focusing in graphene. *Nat Phys* **9**, 225–229 (2013).

11. Jung, J., DaSilva, A. M., MacDonald, A. H. & Adam, S. Origin of band gaps in graphene on hexagonal boron nitride. *Nat. Commun.* **6**, 1–11, DOI: 10.1038/ncomms7308 (2015).

12. Trambly de Laissardière, G., Mayou, D. & Magaud, L. Localization of Dirac Electrons in Rotated Graphene Bilayers. *Nano Lett.* **10**, 804–808, DOI: 10.1021/nl902948m (2010).

13. Moon, P. & Koshino, M. Electronic properties of graphene/hexagonal-boron-nitride moir\'e superlattice. *Phys. Rev. B* **90**, 155406, DOI: 10.1103/PhysRevB.90.155406 (2014).

14. Gorbachev, R. V. *et al.* Detecting topological currents in graphene superlattices. *Science* (2014).

15. Komatsu, K. *et al.* Observation of the quantum valley Hall state in ballistic graphene superlattices. *Sci. Adv.* (2018).

16. Sui, M. *et al.* Gate-tunable topological valley transport in bilayer graphene - Nature Physics. *Nat. Phys.* **11**, 1027–1031, DOI: 10.1038/nphys3485 (2015).

17. Li, Y., Amado, M., Hyart, T., Mazur, Grzegorz. P. & Robinson, J. W. A. Topological valley currents via ballistic edge modes in graphene superlattices near the primary Dirac point - Communications Physics. *Commun. Phys.* **3**, 1–7, DOI: 10.1038/s42005-020-00495-y (2020).

18. Yin, J. *et al.* Tunable and giant valley-selective Hall effect in gapped bilayer graphene. *Science* **375**, 1398–1402, DOI: 10.1126/science.abl4266 (2022).

19. Aktor, T., Garcia, J. H., Roche, S., Jauho, A.-P. & Power, S. R. Valley Hall effect and nonlocal resistance in locally gapped graphene. *Phys. Rev. B* **103**, 115406, DOI: 10.1103/PhysRevB.103.115406 (2021).

20. Zhou, J. & Charlier, J.-C. Controllable spin current in van der waals ferromagnet fe_3gete_2. *Phys. Rev. Res.* **3**, L042033, DOI: 10.1103/PhysRevResearch.3.L042033 (2021).
